# The experiences of doctors in Lesotho during the COVID-19 pandemic: A qualitative study

**DOI:** 10.1371/journal.pone.0350935

**Published:** 2026-06-16

**Authors:** Laura Holdsworth, Mike Jackson, Lucy Piggin, Muila Kambulandu

**Affiliations:** 1 North Wales Clinical Psychology Programme, Bangor University, Bangor, United Kingdom; 2 Family Medicine Specialty Training Programme, Lesotho-Boston Health Alliance, Leribe, Lesotho; University of Auckland, NEW ZEALAND

## Abstract

The COVID-19 pandemic dramatically increased pressure on healthcare staff who were trying to manage and adjust to the novel situation. For many healthcare workers, this came at a cost to their wellbeing. However, as research is predominantly focused on the Western world, less is known about the experiences of healthcare staff in places such as Africa. Using qualitative interviews, this study investigated the experiences of seven doctors in Lesotho regarding their wellbeing during the pandemic. Through interpretative phenomenological analysis, five themes were identified: under pressure, unprepared and out of control; constant uncertainty and threat; isolation and connection; attempts to cope independently and new perspectives. These results indicate that these doctors experienced multiple challenges whilst working during the pandemic which extended beyond the work environment and negatively impacted their wellbeing. Despite this, they experienced support from friends, family and colleagues, found ways to support themselves, and found points of learning from the experience. Implications for how doctors are supported at a national and local level are discussed. Further research should continue to explore doctors’ wellbeing in relation to any long-term implications of the COVID-19 pandemic and during more typical work.

## Introduction

Across the globe, the COVID-19 pandemic increased pressure on healthcare systems, affecting their functioning and ability to deliver quality care [1–3]. As the world attempted to comprehend and manage the virus, it was healthcare staff who faced the day-to-day challenges of managing the condition, treating patients and dealing with the vast loss of life. Research into the pandemic’s impact on healthcare staff has found higher burnout rates, lower morale, poorer mental health or wellbeing, and increased worry [4,5]. A qualitative study of 1379 frontline UK and Irish doctors [6] found themes of feeling exposed, unprotected, overstretched and under-resourced, inhumane care, uncertainty, constant changes and others. Another study [7] identified that 44.2% of the 5440 UK and Irish emergency, anaesthetic and intensive care department (ICD) doctors who completed the General Health Questionnaire-12 met criteria for ‘psychological distress’.

Research has suggested an association between healthcare staff’s poorer wellbeing and pandemic-related factors such as shortage of resources, worry about infection and exposure to the disease [8–10]. Often healthcare staff experience increased feelings of pressure resulting from increased workload, and difficulties adapting to changing protocols and new PPE, caring for unwell colleagues, and caring for seriously ill patients [11]. This raises significant concerns regarding the wellbeing of healthcare staff, how they have managed, or not managed to care for themselves whilst working to care for others.

This issue of poorer wellbeing in healthcare staff may be particularly concerning in low- to middle-income countries such as Lesotho, where the factors associated with poorer wellbeing are more likely to be present. Results from one qualitative study in Lesotho found that healthcare staff experienced an increased workload, stigma and discrimination, and negative emotions in the early stages of the pandemic when fear of infection, death rates and lack of knowledge was high [10]. An online survey study in Ghana [12] found that staff’s fear of infection was high and that they felt unprepared to manage the pandemic. These factors were associated with stress and burnout in staff. In another study [13] questionnaires administered to 171 nurses in Nairobi revealed that almost 50% of respondents were experiencing depression, burnout and/or anxiety.

The Kingdom of Lesotho has a population of 2.3 million (2023) [14]. The government spends 11.1% (comparable estimate) of its total expenditure on health [15], with 1.8% of its health budget spent on mental health [16]. 11.1% is a figure higher than the continent’s average (7.3%), but lower than neighbouring South Africa’s 15.3% and the UK’s 19.5% comparable estimates [15]. The presence of appropriately trained healthcare staff is low. Data indicates that there are only 4.73 medical doctors per 10,000 people (in 2018); compare this to the UK where there are 30.04 per 10,000 (in 2020) [16]. The overall picture in Lesotho is one of a low resourced healthcare system serving a population with high health needs and thus raises concerns about staff wellbeing over the pandemic.

Lesotho has connections with Wales. The charity Dolen Cymru Lesotho Link works to enable sustainable projects, partnerships and relationships for the benefit of communities in Lesotho and Wales. The Betsi-Quthing Partnership Health Link supports healthcare development in Lesotho. During the COVID-19 pandemic, Clinical Psychologists from Betsi Cadwaladr University Health Board provided virtual staff wellbeing support to family medicine trainee doctors. Therefore, these links provided the opportunity for intercultural research exploring medical staff’s experiences during the pandemic. With limited research into this geographical area on the topic of medical staff’s wellbeing during the pandemic, and even less indicating how healthcare staff coped during this period, an explorative approach is required to develop an in-depth understanding of this experience. This research asks how do doctors and nurses in Lesotho understand and manage their wellbeing in the context of COVID-19? Through exploring this, it is hoped that a better understanding of the experiences of doctors and nurses can be developed to inform policy and practice in staff wellbeing support. Whilst this research involved both doctors and nurses, this paper will focus on the findings from the doctors’ data. A separate paper will address the findings from the nurses’ data.

## Method

### Participants and recruitment

Seven medical doctors from four districts in Lesotho participated in the research (see Table 1). Mean age was 36 (range 31–48) and mean number of years as a doctor was 7 (rounded to the nearest year; range 2–15). All but one of the doctors were Basotho (from Lesotho); the non-Basotho doctor came from another sub-Saharan African country. All doctors were working in government facilities in Lesotho before, during and after the COVID-19 pandemic. Participants were recruited using purposive sampling; qualified doctors working in Lesotho during the COVID-19 pandemic with an ability to speak English to a standard enabling them to participate in the interview and without any major mental health concerns were eligible to participate. With the support of two charities working across Lesotho and Wales, senior medical professionals in one hospital, one clinic and Lesotho’s family medicine training programme were approached to seek their support in recruiting participants. Initial emails introducing the research aim and methods were followed up with face-to-face meetings led by the first author to provide further information. These senior professionals then identified staff who were eligible for recruitment. Between the 11^th^ and 18^th^ of November 2022, face-to-face meetings with these individuals were arranged to discuss their participation; they were provided with verbal and written information about the research (aim, methods, data use, right to withdraw, benefits and possible risks) and invited to ask any questions prior to participation. Seven doctors chose to participate, and interviews either took place immediately or a later time, depending on the participant’s preference.

**Table 1:** Participant demographics.

Participant	Age Category	Length of Time in Profession (Years)
**1**	30-39	0-5
**2**	30-39	0-5
**3**	40-49	11-15
**4**	30-39	0-5
**5**	30-39	6-10
**6**	30-39	0-5
**7**	30-39	0-5

### Procedure

This study took a qualitative design. Interpretative Phenomenological Analysis (IPA) [17] was utilised due to its focus on subjective phenomena and the lived experience of participants. The intercultural nature of this research meant IPA would be particularly advantageous in supporting cultural humility due to its social constructionist stance. Its use of the double hermeneutic encourages researchers to discover and examine their own assumptions and interpretations [18]. IPA explicitly acknowledges the role of the researcher within the interpretation of the data allowing for recognition that the differing cultures of researchers and participants will have an impact on the results.

A semi-structured interview guide was created and reviewed by the research team. The initial protocol and versions of the interview guide focused on exploring stress, but this was replaced with the word ‘wellbeing’ to allow for a broader and less researcher-led exploration of doctors’ experiences. The guide was developed and structured to allow for participants to settle into the interview and think about changes to the job as a result of COVID-19. It then explored topics relating to the research question, including what the word “wellbeing” meant to participants and any positive or negative impacts COVID-19 had on their wellbeing. Interviews were conducted by the first author, in Lesotho, at participants’ places of work. Due to the potentially distressing nature of the interview topics, it was planned for the first author (a trainee Clinical Psychologist) to utilise her clinical skills to support interviewees experiencing distress in the first instance. Senior medical professionals in the different districts were consulted as to where interviewees could be signposted to should they require further support; approaching mental health staff within the local hospitals was advised as the only option available.

Semi-structured interviews were conducted on a one-to-one basis in a private room at a location convenient to the participant. As per IPA tradition, the interview guide was used flexibly, with participants encouraged to explore aspects of experience that they felt were interesting or important. At the end of the interview, basic demographic data and information regarding the participant’s wish to review the results and/or final article prior to the report being submitted was collected. The mean duration of the interviews was 73 minutes (range 44–92). Interviews were conducted in English, audio-recorded and transcribed verbatim (with significant non-verbal communication such as laughter and long pauses) by the first author.

During the study preparation, interview and data analysis stages, a reflective log was kept by the first author to support bracketing of assumptions and ownership of one’s own perspective. The fourth author, a Lesotho-based researcher and medical doctor, acted as a cultural consultant during the study, particularly in relation to the cultural appropriateness of the interview guide, interview procedure and data analysis.

### Ethical considerations

Ethical approval was granted by the School of Human and Behavioural Sciences ethics committee at Bangor University, Wales, UK (Ref: 2022–17164) and by the Ministry of Health’s Research Ethics Committee, Lesotho (Ref: ID 190–2022). Verbal consent to undertake this research within specific medical facilities was provided by senior medical professionals within those facilities. Participants provided written consent to participate in the research, and were reminded of their right to withdraw and confidentiality prior to interviews commencing.

### Inclusivity in global research

Additional information regarding the ethical, cultural, and scientific considerations specific to inclusivity in global research is included in the Supporting Information S1 Checklist.

### Data analysis

Utilising processes illustrated by Smith, Flowers and Larkin [18], each transcript was examined for its descriptive, conceptual and linguistic features pertaining to the study’s aims. These initial exploratory notes alongside the transcript were transformed into experiential statements and then personal experiential themes (PETs). The cultural consultant then reviewed the exploratory notes, experiential statements and PETs providing feedback on the analysis’ cultural appropriateness. Each transcript was analysed as an individual piece with the first author bracketing off outcomes from previous transcripts. Finally, the PETs were analysed to form group experiential themes (GETs). As suggested by Smith, Flowers and Larkin [18] a GET was only included if it consisted of data from at least four participants.

Four of the seven participants had opted to review the results for accuracy and cultural appropriateness before the report was finalised. The results were emailed to them, however no participants responded, and it was assumed there were no concerns regarding the results.

### Epistemological approach

A constructivist stance was adopted during this research, which assumes that knowledge is developed within a person’s own context through interactions with the world. This has close links with hermeneutic phenomenology which is the basis for IPA and aims to understand a person’s lived experience in a particular context through their interpretations of these experiences. This is appropriate for intercultural research whereby individuals’ experiences are seen to shape their understanding and interpretation of subsequent experiences. This was important for this research as it was desirable to avoid imposing Western constructs onto the participants. Through taking a constructivist approach, the authors were able to question and consider various elements of the research process and their acceptability for the participants. For example, the construct of ‘wellbeing’ was not assumed to have the same qualities across cultures and between individuals, therefore part of the interview involved exploring with participants what their meaning of the word ‘wellbeing’ was.

### Reflexive statement

The first author, a female, white British, trainee Clinical Psychologist with a Psychology BSc, conducted the interviews and data analysis. Concerns regarding being a cultural and racial outsider, and power imbalances in relation to this, were managed through discussions with the cultural consultant, other professionals who had worked in Lesotho, and spending time in Lesotho familiarising herself with the culture and healthcare system. Her previous work in Sub-Saharan Africa created assumptions about the nature of the results. These included an expectation that familial and community relationships would be seen as key to coping during the pandemic, that any mental health problems would be minimised due to fear of stigmatisation, and that staff would be experiencing stress due to a difficult work environment. As per the IPA approach, these assumptions were reflected upon and bracketed off prior to conducting the interviews and analysis [18].

Analysis was led by the data; however, the first author’s individualistic cultural background and mental health training are likely to have influenced the results. Whilst this is acknowledged as an inevitable part in the IPA process, the use of reflexivity, bracketing, and consultation with the cultural consultant were vital in minimising the impact. To counteract the first author’s predisposition to focusing on psychological phenomena, she made a conscious effort during interviews to ask follow-up questions about other phenomena when they naturally arose, and during analysis to consciously avoid dismissing non-psychological phenomena (for example, the physical impact of stress).

## Results

Five GETs were identified across the participants’ interviews Table 2. It was noted that within each theme there was a sense of change and development over time as the pandemic progressed. For example, participants talked more about feeling out of control during the onset and main part of the pandemic, whereas new perspectives developed as the pandemic progressed and eased.

**Table 2:** Contribution of each participant to the group experiential themes and subthemes.

Group Experiential Theme	Subtheme	Participant						
		1	2	3	4	5	6	7
Under pressure, unprepared and out of control	Chaos		✓	✓	✓			✓
	Out of control, helpless and unprepared		✓	✓	✓	✓	✓	✓
	Expectation and pressure			✓	✓	✓	✓	
Constant uncertainty and threat		✓	✓	✓	✓		✓	✓
Isolation and connection	Isolated and alone	✓	✓	✓		✓	✓	✓
	Abandoned by the government		✓		✓	✓	✓	
	Support from others	✓	✓	✓	✓	✓	✓	✓
Attempts to cope independently		✓			✓	✓		✓
New perspectives	Changing Perspectives on what it means to be a doctor		✓	✓	✓			
	Connection with own mortality			✓	✓			✓
	Changing perspectives on self and life		✓	✓	✓	✓		✓

### Under pressure, unprepared and out of control

#### Chaos.

Participants’ descriptions of working during COVID-19 painted a picture of chaos in which there was a lack of preparation, resources (such as PPE, oxygen and human resources), and knowledge about how to treat COVID-19. There was fear due to feeling more exposed to the virus and a sense of disorganisation within the hospitals:

In that time no one knew what was going on, no one knew what should be going on, how we go about it, how we help people, it was all a blur, and… it felt like, um, guidelines were just changing on a daily, today we do this, tomorrow we don’t do it anymore. (Participant 7)

Here we can see Participant 7 stress the lack of knowledge in multiple areas. Language used by participants illustrates the chaos and disruption caused by COVID-19. Participant 4 described the pandemic as an ‘*elephant’*, ‘*battle’, ‘war’* and a *‘continuous storm*’ (Participant 4) conjuring images of power, fear, and widespread destruction. For Participant 7, ‘*COVID was like this, [chuckle] mystical creature right, with a sword, just waiting for you’* (Participant 7). In this image, the unknown nature of COVID-19 is brought to life as a mysterious creature, lurking and threatening everyone’s life. It demonstrates a palpable fear, but also that the lack of knowledge about COVID-19 contributed to that fear.

#### Out of control, helpless and unprepared.

On a personal level many participants described feelings of being out of control or helpless, particularly in relation to seeing patients deteriorate and die. This has strong links with the previous subtheme, where the lack of resources and knowledge meant doctors were unable to provide patients with suitable treatment.

Participants experienced a disconnect with the purpose of their job role, which threatened their identity as a doctor and resulted in moral injury, as demonstrated by Participant 3, *‘we are seeing people who are dying like every day. It’s like… you can’t help people… you were asking yourself questions, am I really a doctor or not?’* (Participant 3). Given that patients dying is not a new experience for doctors, this reaction suggests that participants felt helpless in a novel way and suffered moral injury through witnessing deaths.

The lack of control spread across various areas of their life and work. Participant 7 expressed thoughts in relation to not being able to control the potential personal impact of the pandemic:

I mean other diseases, let’s take hypertension, let’s take diabetes... I see people who have it, but also I’m thinking… it’s a secondary condition. If we try and look after ourselves, … these are less likely to develop right. But with COVID it wasn’t… anything extra that you had to do, all you had to do is breathe, which we all do… the sure way of really protecting yourself to say, yes, this will help, I do not have that... So, that for me was, we don’t have control over this one, yeh. (Participant 7)

Other participants spoke about how they felt helpless or lacking control in their relationships:

In terms of, uh, my wife… I felt useless… I thought that what I had promised, I am not fulfilling, and her fears in marrying a doctor, was not going to give her time and everything, were now becoming manifest. So, I felt useless at that point, and the more I try hard to keep up with the requirements of the family, the more I was getting exhausted. (Participant 2)

Here we can see the impact of COVID-19 extending beyond its immediate vicinity of clinical work. The exhaustion from work had impacts on personal relationships, and resiliency was reduced as doctors attempted to deal with the situation unfolding in front of them. Participants found themselves questioning their existence, autonomy, and identities in life. These elements are explored further in the theme, ‘new perspectives’.

#### Expectation and pressure.

Despite the experiences described above, doctors still felt an expectation and pressure to continue working despite the lack of knowledge and resources. This expectation stemmed from the external pressures to continue working and self-imposed internal expectations.

I was part of the team that went out to… the second recorded case of COVID… You’re just being grabbed like, okay, we have to go. It’s like ah, okay, I’m going [pause] but, have you even considered that we are talking about a person who has been diagnosed with COVID? What do I have to wear? Prepare me mentally, prepare me psychologically, because this is also something new to me. Nothing is being done. (Participant 5)

Participant 5’s description demonstrates the pressure put on her, with no consideration for how prepared she felt. The use of the word ‘grabbed’ suggests a lack of choice in the decision, thus creating a situation for moral injury. In contrast, Participant 3 spoke about his internal sense of obligation and expectation to continue working.

Many colleagues got it [COVID-19] and it make me like to stop working [chuckle] but it was not easy. At the same time, you were saying, “no I am a doctor, I have to help people”, that duality in, in thinking, it was not easy, what should I do? But at least we are doctors we, we try to do our best. (Participant 3)

Here we witness Participant 3’s internal dilemma between his fear of contracting COVID-19 at work and his sense of duty as a doctor. There is a split between his sense of self as a doctor and sense of self as a person, as evidenced by his use of the world ‘duality’. Again, this appears to create a situation where moral injury could occur.

### Constant uncertainty and threat

Participants’ wellbeing was impacted by the uncertainty and threat from COVID-19 which were experienced as constant, inescapable, and ‘*terrifying’* (Participant 7). The uncertainty surrounding COVID-19 made the threat of death even more powerful, causing ‘*dread’* (Participant 2) and ‘*fear’* (Participant 6), and penetrating across time. ‘*The stress was like, you don’t know what’s going to happen to you the next day. The future, you don’t know the future, what’s going to happen in future about yourself, about your family. That was eh the stress*’ (Participant 3). Participant 7 felt similarly:

After the pandemic hit, it was a scary time… Um, I mean in that time we really should dance with death a lot, right… I feel like we met face-to face often times and so the reality of it really was more heightened. (Participant 7)

Participant 7’s fear of being ‘face-to-face’ with death; seeing healthy, young people die made the risk of death feel even more uncertain and that it could happen to anyone. *‘With COVID, it had no respect for health or ill health, age, you know, everyone was just game’* (Participant 7). The use of ‘dance with death’ and ‘face-to-face’ highlights the closeness that she experienced with death during this time; she was confronted with it, trying to make the right moves to delay the inevitable.

### Isolation and connection

#### Isolated and alone.

For six participants, the experience of isolation and aloneness were themes in their interviews. Isolation occurred in the workplace and home, both physically and psychologically. Teamwork was lost as healthcare staff were assigned to either the COVID-19 ward or not. The increased work stress and exhaustion had an impact on participants’ personal lives. Relationships became more strained, and this created an unhelpful spiral of worsening wellbeing, as described by Participant 2, ‘[you] *don’t have time for your friends anymore, and now they say you have changed. So, you don’t have your support structure at that point’* (Participant 2).

For many doctors, they were already living alone before the pandemic. This physical isolation left them struggling to manage their wellbeing and feeling psychologically alone, as they had no support after work. Participant 5 describes this:

When you go home, you need someone to talk to [pause]. Someone who’ll just give you a hug… and say, it will be okay. [pause]. Someone you can just talk to about your stress, who’ll guide you and give you advice, you know, someone who’ll just listen, and I didn’t have that (Participant 5)

Despite the distress at being alone, some participants also noted positives in the protection it afforded their family who were not in physical contact with them:

I live on my own right, so I wouldn’t visit my parents after having gone for my shifts because I was worried if I had the condition and so I didn’t want them exposed… which really made the whole isolation thing so much easier. (Participant 7)

#### Abandoned by the government.

The aloneness and isolation experienced by the doctors was partially created or exacerbated by the lack of support from authority figures and the government. Participants felt that the government and their superiors did little to support them during the pandemic, instead abandoning them to ‘*fend for themselves*’ (Participant 2). Participants reported feeling unappreciated by the government:

So, there was no sequential way of uh giving information out, uh, from our ministry unfortunately. So, it affected us and how we are supposed to work, and there was a lot that we’re looking at from government to, to support us to make it easier for us to work… but there wasn’t anything. You’re frustrated at the end of the day. (Participant 5)

Participant 5’s frustration links to an expectation of support and advice from the government, a sense of seeking for leadership and information, but ultimately finding nothing. Participant 2’s description of the action healthcare staff took to gain support from the government demonstrates the severity of how abandoned and let down healthcare staff felt; ‘*we had to force them. We forced them to give us PPE, we forced them to give us, eh, the risk allowance, and it wasn’t even enough, and they didn’t even pay it in full’* (Participant 2). Participant 2 refers to ‘the risk allowance’, a payment made in recognition of the increased risk healthcare staff were experiencing. Here we can see that he felt unappreciated by the government because the amount was little compared with the risks he was experiencing; healthcare staff’s lives were worth so little to the government.

#### Support from others.

There was the sense that family, friends and sometimes colleagues, partially made up for the gaps left by the authorities. Support from others was essential for the doctors in managing their wellbeing:

There were those periods that we’ll feel that, no, man I cannot take this anymore… However, the fact that the social, emotional aspects there, where those individuals that are significant to us who were at least closing that gap, it made us not to crash. (Participant 4)

Participant 4’s ‘significant’ others clearly helped him to keep going. Without these people his wellbeing would have deteriorated to a point where he ‘crashed’, giving an image of him being damaged and destroyed by the pandemic’s impact. There was still a gap between how his wellbeing was and where it is typically, which refers to the need for further professional support.

### Attempts to cope independently

Whilst they were abandoned by authorities, and experienced isolation and aloneness, participants were able to turn towards themselves for support. Some doctors turned to religion, others to managing their physical health and some to emotion management strategies. Participant 5 described a journey of exploration in coping independently, sometimes reaching out for ways to cope which were ultimately not successful:

Sometimes you find yourself locking yourself in this office, you cry, you go home, you’re only thinking can I get something that can sedate me, you start [pause] drinking, unnecessarily, just to numb yourself, just for something that will make you not to think about this environment that you’re working in… I found myself doing things that I never had anticipated that I would do, just because I’m trying to relieve myself of the stress. (Participant 5)

This extract shows Participant 5’s attempts to distract herself from the difficulties and emotions. Desperation is felt in her attempts to do anything to cope, reaching out to things she never thought she would. However, some doctors described more success in self-care:

Something that I do not have control over [the death of a patient], yes I may feel that guilt, but… it’s a feeling and feeling is not um, the reality of what is going on, but now I had to understand that… and separate the feeling from the realities, yes, of life. (Participant 4)

Here Participant 4 describes balancing his emotions with rational thinking. Recognising that emotions are not necessarily a reflection of reality helped him to maintain his wellbeing.

Overall, relying on oneself during the pandemic was a key part of managing wellbeing, however the methods used varied between participants with varying degrees of success.

### New perspectives

Despite the themes above, participants also spoke about the learning gained from working as a doctor during the pandemic. This impacted both their professional and personal lives. This view of the pandemic, as a time of learning and development, could be seen to mitigate the negative impact on wellbeing, as all the distress and loss were not for nothing.

#### Changing perspectives on what it means to be a doctor.

Participants spoke about a deeper connection with patients and a recognition that as a doctor they can work more holistically, seeing the person as someone with a context to take into consideration, and needs beyond just the physical. For example:

That is where now my eyes open, to see you are not just going to focus on the disease process here, but you need to also take care of other significant um, aspects of a patient, like psychological ones. Some of them would need the spiritual support. (Participant 4)

Participant 4’s increased awareness of the different facets of healthcare came about from witnessing fear in patients. It exposed him to their psychological and spiritual needs, and in a low resource setting, he was the only one available to help. For participants, the pandemic was seen as a chance to implement previous learning and a reminder that their learning did not stop at the end of their medical training.

#### Connection with own mortality.

Experiencing the vast impact and indiscriminate nature of COVID-19, how ‘*it can affect anyone… it’s not that because you know more about it, it’s not going to progress towards* [death]’ (Participant 7), appears to have led to some participants connecting with their own mortality in a new or more profound way. Participants recognised that they are ‘*vulnerable also’* (Participant 3); COVID-19 had just as much chance of affecting them. This was a scary reality check for participants who were working with COVID-19 patients, often without adequate protection.

#### Changing perspectives on self and life.

The threat of death, connection with own mortality and the distress caused by the pandemic led to reflections on the doctors’ personal lives. For example:

you realign yourself again to see really what is important, you always are busy, you don’t have a lot of time to see your parents, because oh, I’m at work, so now in that time, it was, is it really worth it? Right. So you redefine that again, so you kind of have people who… you just never knew just how important they were until they fell ill… I took more time to check up on people, to find out how they are doing. (Participant 7)

Participant 7 and others reflected on decisions in their lives which have taken them away from their family and the people they want to be. Participants discussed changes they wanted to make to be more engaged with the things they value; the pandemic was the spark which ignited this way of thinking. For other participants their changed perspectives related more to the world in general. Participant 4 found that his experiences during the pandemic taught him the importance of preparation. In his words there is sense of trepidation and a new perspective of life as unstable:

COVID-19, it taught me, it taught us, and taught the whole world that the world is not a stable place… we need to always be prepared for anything. Because the consequences of not preparing is, if some of these things come and hit us, we end up finding ourselves in a despair. (Participant 4)

## Discussion

Results indicate that these doctors, working in Lesotho during the COVID-19 pandemic, experienced a deterioration in their wellbeing evidenced by isolation, aloneness, abandonment, and fear relating to uncertainty, lack of control, pressure, and helplessness. However, self-care and connection with others provided an antidote. Experiences also led to new perspectives on life.

COVID-19 was experienced as an immense and dangerous entity instilling fear and creating an uncertain, chaotic environment. Other research has reported similar results; during the pandemic staff felt unprepared [12] and some of the main challenges to healthcare workers were related to preparedness and availability of resources [10,19]. Research [19] also found that uncertainty related to concerns about how the pandemic would progress, how to manage COVID-19, and personal safety.

For staff in low- and middle-income countries, exposure to the virus may have felt particularly threatening due to witnessing deaths across high-income countries with better resourced healthcare systems. For example, the frequent lack of access to safe hand washing facilities found across sub-Saharan Africa [20] puts healthcare workers at higher risk. There are concerns for the wellbeing of staff in Lesotho, as evidence indicates that increased contact with COVID-19 patients without adequate protective resources, lack of knowledge in how to manage the condition, and a lack of other resources are associated with worse mental health in healthcare workers across the globe [21,22].

The terrifying experience of feeling out of control, helpless, and unprepared was felt to be unrecognised by others who expected doctors to continuing working in high-risk environments. This is reflected in other research [23] which found that, across the world, some healthcare workers have felt “coerced” into working with patients or in unsafe conditions. Culturally, the Basotho value their elders, seeing them as wise and knowledgeable, with an important role in education and leadership [24]. Whilst this predominantly relates to familial and societal issues, it may still have been difficult for younger Basotho doctors to go against expectations to continue working. Additionally, there are still significant gender inequality issues in Lesotho. For example, girls/women aged 15 and above spend 15.6% of their time on unpaid care compared to 6.2% for their male counterparts, and in 2018, 16.5% of girls/women aged 15−49 reported having experienced physical and/or sexual violence within the past year from a current or former partner [25]. Whilst data indicates some improvements for Basotho women (50.1% of girls complete lower secondary education, compared to 33.6% of boys, and the percentage of women who own land equals that of men [26], gender inequality still plays a role on how empowered women in Lesotho are. This inequality may have led to female doctors being disproportionately affected by the COVID-19 pandemic and reduced their ability to advocate for themselves within the workplace. Psychological safety may play an important role for both younger and/or female doctors; they may have felt unable to raise their safety concerns due to apprehensions of not being heard or personal negative consequences. This links to the participants’ feeling abandoned by the government. Continentally, there are public beliefs that political institutions cannot be trusted due to corruption [27,28] and during the pandemic there was a lack of transparency and accountability across many African countries regarding how COVID-19 funds were used [29]. This provided a ready-made narrative for how the doctors could understand their experiences during the pandemic and likely decreased their psychological safety. In other research [30] leadership style played an important role in levels of psychological safety; dominant leaders decreased psychological safety, and so for doctors in Lesotho, the pressure and expectations experienced from senior officials may have further decreased psychological safety.

As psychological safety is important for increasing helpful learning behaviours, performance, effective teamworking, and creative problem solving [31,32], it is plausible that a lack of psychological safety could have exacerbated the feelings of chaos and unpreparedness, creating a barrier to staff being able to make changes in helpful and safe ways. The experience of pressure and lack of psychological safety is likely to have led to moral injury, with participants feeling forced into engaging in actions which go against their own morals. This term, although of Western origin, has relevance in Africa where there are already local practices aimed at healing the impact of moral injury [33].

However, there were also internal expectations from the doctors themselves; a sense of duty and obligation to continue working to save other people’s lives. This was not an uncommon experience during the pandemic, as healthcare staff often experience a dilemma between feeling committed to helping patients, but also wanting to protect themselves and their families [19,23]. When considering medical ethics and morality, particularly the deontological practice of doing no harm, as pledged during the Hippocratic oath, and the very personal nature of the situation, wherein doctors knew that their own and their loved ones’ lives were at risk, it is understandable that this experience led to psychological distress. Many doctors join the profession due to wanting to care for and help others; holding this value may have worsened this dilemma for them. The role of social identity can further enrich the understanding of why this dilemma of treating patients versus protecting themselves was so difficult. Participants’ use of language in identifying as a doctor indicated that this social identity was important to them. Equally, some participants discussed their Christianity, which served to both increase their sense of control (e.g., through praying) and accept that aspects are out of their control (i.e., that ultimately the outcomes are in God’s hands). Choosing to treat patients rather than prioritise their own safety may have been an attempt to remain within their identities as doctors and/or Christians (and the obligations which go along with this), and maintain the status of these groups. For many, the choice to become a doctor originates in part from a personal interest in science, social justice and helping others [34]. This “calling” to pursue a medical career may mean doctors place a stronger emphasis on their identity as a doctor than on other areas of their lives and a stronger desire to protect this identity. Similarly, Christianity emphasises the importance of love for others and helping others. It may be that Christian doctors identify strongly with this aspect of their religion and that this impacted their decisions to continue treating patients during the pandemic.

Despite the isolation and lack of support experienced by staff, support from others and finding ways to cope independently helped the doctors through the pandemic. Other research [23,35] also found that across multiple countries, family and friends were key sources of support during the pandemic. It is well documented that social isolation has deleterious effects on wellbeing [36,37], thus support from others during the pandemic is likely to have been essential in preventing wellbeing from worsening further. As in the current study, a multi-country survey study [35] found a wide variety of coping strategies, many of which involved self-care. Engaging with religion was one of the most common self-care strategies used by doctors [35], and some participants in the current study also relied on this (three doctors spoke about using prayer and/or church as a method of coping, a further two indirectly indicated that prayer formed part of their coping strategies). With 98% of Lesotho’s population identifying as belonging to a particular religion [38] it could be expected that more of the doctors would have identified religion or religious practice as a coping strategy. It is unclear why this was not the case, and factors relating to the interviewer’s characteristics, study design and/or sample characteristics may have played a role in this result.

Amongst the challenges of the pandemic, the doctors in this study still found that they gained things from the experience. Most notably, they reported gaining new perspectives on different aspects of their lives. For some participants, the gains achieved may reflect post-traumatic growth, with fundamental shifts in how the world, self, and relationships with others are perceived [39]. Whilst ‘post-traumatic growth’ is a Western term, the concept of positive change following crises is not new and spans the globe [40]. Research has found that post-traumatic growth occurs in work settings involving exposure to trauma, but that the organisation’s response to the exposure impacts post-traumatic growth [40]. This raises concerns for the opportunities for post-traumatic growth for the doctors in this study given the lack of organisational support received.

### Implications

Whilst the current study was not designed to be generalised, research across Africa has found similar results in relation to healthcare staff’s wellbeing being negatively impacted during the pandemic [41–43]. The current research suggests that those leading Lesotho’s healthcare system should provide more wellbeing support to staff, particularly in relation to how leaders and the government communicate with, value, and protect healthcare staff. This study also advocates for the provision of mental wellbeing resources for healthcare staff.

As the COVID-19 pandemic has eased, it is important that research continues to investigate the wellbeing of doctors in Lesotho to gain a better understanding of any long-term implications of the COVID-19 pandemic and doctors’ wellbeing during more typical work. Gender differences were not explored within this research, and given the persistent gender inequality within Lesotho and across the world, this is an important area of investigation to better understand the impact of gender inequality on doctors’ experiences.

### Limitations

All interviews were conducted in English, one of the official languages of Lesotho, but less frequently used than Sesotho. Whilst all doctors were able to communicate in English to a high level and some would have trained in an English-speaking context, this may still have limited their ability to fully express themselves. The interviews may have elicited even richer data had they been conducted in Sesotho. However, translation comes with its own limitations; finding appropriate translations for metaphors or words without a direct translation, and the translator’s decisions on how to translate words, are just two examples of how translation can alter the meaning of a person’s words [44,45]. Furthermore, the use of English may have magnified any power differences between the interviewer and participants. Whilst this research aimed to be culturally sensitive, the use of English can be seen as a version of colonialism within research.

Similarly, the majority of psychological models, concepts, and narratives have been developed in Western contexts. Whilst efforts were made to analyse and interpret the data in a culturally sensitive manner, it cannot be ignored that Western narratives dominate, and it is impossible as a researcher from a Western population to completely detach oneself from these narratives. The use of IPA helped to keep this in mind and acknowledge that the first author has given a different perspective to the data than may have been achieved with a Basotho researcher. The inclusion of the cultural consultant helped develop cultural appropriateness during data analysis.

## Conclusion

This research has highlighted the challenging experiences of doctors in Lesotho during the COVID-19 pandemic, finding five themes representing how these doctors experienced and coped with their wellbeing. The results paint a concerning picture, illustrating stress, worry, threat, and isolation. However, there were positives during the pandemic; a recognition of how participants’ wellbeing was supported by others and themselves, and how their experiences have led them to gaining new perspectives on life. It is imperative that governments and organisations invest in supporting doctors, whilst future research continues to investigate how we can better understand the needs of healthcare staff in Lesotho.

## Supporting information

S1 Checklist Inclusivity in global research. (DOCX)
