# Supplementary material for: The experiences of doctors in Lesotho during the COVID-19 pandemic: A qualitative study
Source: PLoS One. 2026 Jun 16;21(6):e0350935. doi: 10.1371/journal.pone.0350935 (PMC13271460; doi:10.1371/journal.pone.0350935)
Supplement: Attachment — Submitted filename: Response to Reviewers.docx [file pone.0350935.s002.docx]

North Wales Clinical Psychology Programme

Bangor University

Brigantia Building

College Road

Bangor

UK

LL57 2DG

PLOS ONE

Dear Rosemary and Reviewers,

**Re manuscript:** Intercultural understandings: The experiences of doctors in Lesotho during the COVID-19 pandemic

Thank you for taking the time to review our article, and for your thoughtful feedback and helpful advice. Below I have responded to each point raised from the review process.

From editor:

1. I believe that the document meets PLOS ONE’s style requirements. Within the manuscript the changes made were to the corresponding author information, moving the title of the table and removing the italics, and formatting the “acknowledgements” and “references” titles.
2. I have now completed and attached the questionnaire on inclusivity in global research.
3. Regarding data restrictions, Lesotho is a small country with a small number of doctors. Additionally, some of the doctors interviewed work in specialist medical centres (of which there may be only one in the country). I believe that if someone had access to the data set, then it would be possible to identify some of the participants, particularly those who have distinctive demographics, or discussed distinctive experiences during their interviews. For this reason, it has been decided not to make any of the data available for others to use. This was decided by the research team at the study design phase. The relevant ethics committee to contact regarding data access issues is: School of Human and Behavioural Sciences Ethics Committee, Bangor University, 43 College Road, Bangor, Gwynedd, LL57 2DG, UK. Phone - +44 1248 382205. Email – ethics@bangor.ac.uk
4. Full ethics statement and informed consent information has now been provided within the Methods section.
5. I have checked the reference list and it appears to be complete and free from retracted papers.

From reviewer 1:

1. Towards the end of the introduction I have included a sentence indicating why there is the need for qualitative methodology in this research area. The procedure section details why an IPA approach was used. Further details on why this approach is used would detract from the main aspect of the research and information on why qualitative methodologies and IPA are used can be found from other resources (e.g. Smith et al.’s book on IPA).
2. I have incorporated a table outlining demographic features. I have kept the categories broad to maintain anonymity and did not include medical speciality due to some specialities having very few doctors working in that area.
3. Comments in relation to the use of English instead of Sesotho and the impact that this had on the research process have been addressed by expanding the limitations section. Whilst this could be a much larger discussion point, and indeed the considerations of how translation impacts the research approach is a fascinating one, it was felt that this article is not the best place to address this topic in detail and other articles which discuss this topic have now been referenced.
4. Regarding the request to explore further cultural, gender and religious aspects which relate to this piece of research, I have done so within the discussion section. There are three sections which I have expanded on, one discussing gender inequality, another discussing the social identities as doctors and/or Christians, and a final part on religion specifically. I have aimed to expand further on these areas without increasing the word count too much.
5. Thank you for the additional comments highlighting some grammatical and other errors. The corrections have been made. The population figure has also been updated.

From reviewer 2:

1. Comment from reviewer, no action required.
2. Ethics statement amended as requested.
3. The title has been amended to better reflect the contents of the manuscript.
4. The word “Introduction” has been added as a title after the abstract and before the introduction.
5. The methods section of the manuscript indicated the sampling method, number of districts participants were working in and has been amended to better indicate that from the invited participants, seven chose to participate.

“Cross-sectional” has been removed from the procedures section.

Amendments have been made to better illustrate considerations in the development of the interview guide. The interview guide was not tested for reliability though.

Information relating to ethical considerations is interspersed within the article e.g. information regarding consent, supporting participants if they experienced distress, the option to review the results prior to publishing to check for fair interpretation of their interview.

The process of using IPA to analyse the data is detailed in the data analysis section.

The epistemological section has been amended to correct terminology and better align with the study type and design.

6. Table 2 shows the themes and subthemes developed. Participants names are pseudonyms; the manuscript has been amended to state this (within the “participants and recruitment” section).

The first person has only been used in quotes from participants. Authors are recognised as part of the research process in IPA and so relevant information regarding the authors has been included in the report for this reason.

Yours Sincerely,

Laura Holdsworth

**Contact details**

Corresponding author: Laura Holdsworth

Email: laura.holdsworth@wales.nhs.uk

Phone: +44 3000 840306

Author: Mike Jackson

Email: mike.jackson@bangor.ac.uk

Phone: +44 1248 351151

Author: Lucy Piggin

Email: l.piggin@bangor.ac.uk

Phone: +44 1248 351151

Author: Muila Kambulandu

Email: mkambulandu@leboha.org.ls

Phone: +266 5951 8961

Address: PO Box 0962, Maseru West 0105, MNM Building, Florida, Maseru, Lesotho
