# Supplementary material for: The experiences of doctors in Lesotho during the COVID-19 pandemic: A qualitative study
Source: PLoS One. 2026 Jun 16;21(6):e0350935. doi: 10.1371/journal.pone.0350935 (PMC13271460; doi:10.1371/journal.pone.0350935)
Supplement: Attachment — Submitted filename: Review for Plos.docx [file pone.0350935.s004.docx]

**Name of the Reviewer**: Prof Maseabata Ramathebane

**Study title**: The experiences of doctors in Lesotho during the COVID-19 pandemic: A qualitative study

I find this study very important for Lesotho and other LMIC countries. It highlights the experiences of doctors working in a resource-limited environment. The study employs a robust qualitative methodology. It gives confidence that the results will reliably answer the research question. The study is culture sensitive, but if conducted in Sesotho, it could have yielded more accurate results. However, because the participants were doctors, English was good enough. The results highlight experiences that left a lasting mark on their professional journey, whereby there was a thin line between a doctor and a person who was torn between helping patients and exposing themselves to a killer disease, fearing having the disease and infecting family members. Lack of support that led to isolation. The research highlights the important aspect of the government's lack of support, including limited access to PPE and risk allowances.

The study could have had a greater impact if it had drawn on research from Sub-Saharan Africa to compare experiences in similar settings. But the study carefully weighed all these and took steps to address the matter, as it should reflect culture, by including a local person from Lesotho who understands Basotho culture.

All in all, the study is very well written and deserves publication to make it more accessible for future pandemic response in LMICs.
